# Compressive Strength Assessment of Soil–Cement Blocks Incorporated with Waste Tire Steel Fiber

**DOI:** 10.3390/ma15051777

**Published:** 2022-02-26

**Authors:** Joaquin Humberto Aquino Rocha, Fernando Palacios Galarza, Nahúm Gamalier Cayo Chileno, Marialaura Herrera Rosas, Sheyla Perez Peñaranda, Luis Ledezma Diaz, Rodrigo Pari Abasto

**Affiliations:** 1 Department of Civil Engineering, COPPE, Federal University of Rio de Janeiro, Rio de Janeiro 21945970, Brazil; 2 Department of Civil Engineering, Faculty of Technology, Universidad Privada del Valle, Tiquipaya Campus, Tiquipaya MQ9F+GH, Bolivia; fpalaciosg@univalle.edu (F.P.G.); ccn0025217@est.univalle.edu (N.G.C.C.); hrm2014954@est.univalle.edu (M.H.R.); pps0028721@est.univalle.edu (S.P.P.); ldl0029367@est.univalle.edu (L.L.D.); par0028483@est.univalle.edu (R.P.A.)

**Keywords:** scrap tire recycled steel fiber, soil–cement blocks, compressive strength, sustainability

## Abstract

The rapid growth in waste tire disposal has become a severe environmental concern in recent decades. Recycling rubber and steel fibers from wasted tires as construction materials helps counteract this imminent environmental crisis, mainly improving the performance of cement-based materials. Consequently, the present article aims to evaluate the potential use of waste tire steel fibers (i.e., WTSF) incorporated in the manufacture of soil–cement blocks, considering their compressive resistance as a primary output variable of comparison. The experimental methodology applied in this study comprised the elaboration of threefold mixtures of soil–cement blocks, all of them with 10% by weight in Portland cement, but with different volumetric additions of WTSF (i.e., 0%, 0.75%, and 1.5%). The assessment’s outcomes revealed that the addition of 0.75% WTSF does not have a statistically significant influence on the compressive resistance of the samples. On the contrary, specimens with 1.5% WTSF displayed a 20% increase (on average) in their compressive strength. All the tested samples’ results exhibited good agreement with the minimum requirements of the different standards considered. The compressive resistance was evaluated in the first place because it is the primary provision demanded by the specifications for applying soil–cement materials in building constructions. However, further research on the physical and mechanical properties of WTSF soil–cement blocks is compulsory; an assessment of the durability of soil–cement blocks with WTSF should also be carried out.

## 1. Introduction

Steel fiber reinforcement improves the mechanical performance of cement-based materials (e.g., concrete, mortars, blocks, and other cement-based materials). Steel fibers’ usage enhances the mechanical properties of these building materials. To date, the most studied features are the compressive and flexural strength of cement-based materials reinforced with steel fibers. Moreover, their ductility and resistance for dynamic loadings have been analyzed in multiple investigations [1,2]. Past research demonstrated the technical feasibility of steel fibers’ usage in construction materials, particularly incorporated in concrete composite materials [3,4]. However, using reinforcement percentages of steel fibers higher than 1% may raise manufacturing expenses [5,6]. With this in mind, the objective of sustainability in the construction industry might be achieved by utilizing waste tire steel fiber (WTSF) as a green initiative recourse [7].

In the last decade, rapid population growth and land-use changes have generated a significant demand and increase in the vehicles fleet. This situation has led to a gradual increment in tire need and tire waste, yielding a direct impact on the environment [8]. As a result, approximately more than one billion tires are discarded worldwide each year [9,10], with 4.46 million tons [11] belonging to the United States of America. On the other hand, Bolivia generates roughly 1.5 million of tire waste per year [12]. In developing countries, environmental problems are primarily related to lack of planning and inappropriate design of rubbish dumps [13]. Furthermore, these materials’ natural degradation is slow and laborious [14]. Hence, tire recycling in construction materials presents a sustainable alternative, accentuating the re-utilization of rubber [7,15,16] and WTSF [2,6].

The amount of WTSF extracted can represent up to 15% of the total weight of the tires [17]. Moreover, as demonstrated by a few authors, this material possesses mechanical properties analogous to industrial steel fibers. Thus, WTSF has also been investigated as reinforcement in cement-based materials [18,19]. However, recycled steel fibers’ obtention difficulty prevents manufacturing and commercialization on a large scale; the amount of WTSF collected depends primarily on the obtaining process and the tire type. Therefore, additional research is required to determine its influence on the performance of cement-based materials in the long term [9,20].

On the contrary, soil–cement composite material has been widely spread for sustainable construction. Specifically, soil–cement blocks are a low-cost alternative in construction because they require low energy consumption, no combustion process, and no transportation of materials since the blocks can be produced on-site [21]. Additionally, the production of soil–cement blocks can reutilize other solid waste or materials that negatively impact the environment but enhance the brick’s mechanical properties [22]. Various investigations have improved the properties of this material with the use of different natural fibers, such as coconut [23] and palm fibers [24]. In addition, studies of granite cutting residue adjunction (GCR) [21] and recycled tire fibers [25] are also reported; the latter found that recycled steel fibers help absorb plastic energy and resist deformation.

Sukontasukkul and Jamsawang [26] demonstrated that the use of steel and polypropylene fibers improves the flexural performance of soil–cement materials. The authors used percentages less than 1%, where polypropylene fibers presented optimal results. Tajdin et al. [27] used three types of fibers: jute, polypropylene, and steel, to reinforce soil–cement materials. The results showed an increase in the material’s mechanical performance, specifically for the compression, tensile and flexural strength. On the contrary, Bakam et al. [28] used cassava fibers to reinforce earth blocks. A brief literature review has shown that studies on the behavior of soil–cement blocks are still limited and even more limited regarding WTSF, unlike other cement-based materials, such as concrete and mortar, where the use of WSTF is widely studied [29,30,31]. Therefore, using soil–cement with WTSF as a novel construction material could positively impact the environment, thus reducing the consumption of traditional materials and the waste from used tires.

This article aims to assess the potential use of WTSF in soil–cement blocks, centralizing the study on their compressive strength behavior. The latter is considered the primary mechanical feature required by several codes to be accomplished. In addition, a classification of the type of block for use in construction can be obtained by compressive strength achieved [32,33,34,35,36,37]. Hence, an exploratory and experimental campaign was developed where the soil–cement blocks were manufactured with different percentages of WTSF (0%, 0.75%, and 1.50%), verifying their impact on the blocks’ compressive strength.

## 2. Materials and Methods

**Figure 1:**
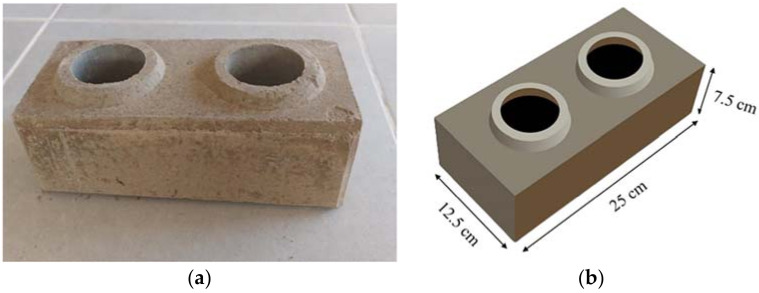
Soil–cement block sample: (**a**) 0.75% WTSF, and (**b**) dimensions.

Soil–cement blocks were prepared, incorporating steel fibers from waste tires (i.e., WTSF) to their matrix composition. Threefold volumetric additions of WTSF were studied: 0% (control), 0.75%, and 1.5%. All compressed earth block samples had 10% of Portland cement added to the mixture (Figure 1) since this cement quantity has demonstrated better mechanical properties outcomes [38,39,40].

**Figure 2:**
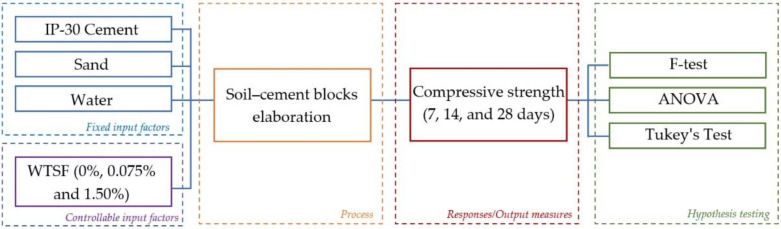
Research methodology.

Figure 2 presents the methodology followed in this study. One controllable input factor (% WTSF) is contemplated; the remaining factors (materials used), although they may be controllable, were kept fixed. This procedure was followed to monitor the influence of WTSF on the compressive strength of soil–cement blocks. The F-test was utilized for the hypothesis test to compare the variance of the means of different levels of factors with the individual variances. The latter was corroborated by a Tukey test, comparing the individual means from the analysis of variance (ANOVA).

Locally available IP-30 Portland cement classified as Type IP Portland Pozzolana cement as per the ASTM C595/C595M-20 [41] was employed. Chemical and physical cement features are detailed in Table 1 and Table 2, respectively.

The soil material employed for the sample’s preparation was extracted from the southern area of Cochabamba, Bolivia, at UTM coordinates 19K 799063.33 E 8070682.40 S. ASTM D422 [42] standard was used to perform the soil’s granulometric analysis. Atterberg limits in the soil sample were determined as per the ASTM D4318 [43] specification. In addition, the soil material was classified following the AASTHO M145 standard [44] recommendations and the unified soil classification system (USCS) [45]. Finally, soil’s optimal moisture content was measured employing the Proctor compaction test as per the ASTM D698 standard [46].

**Figure 3:**
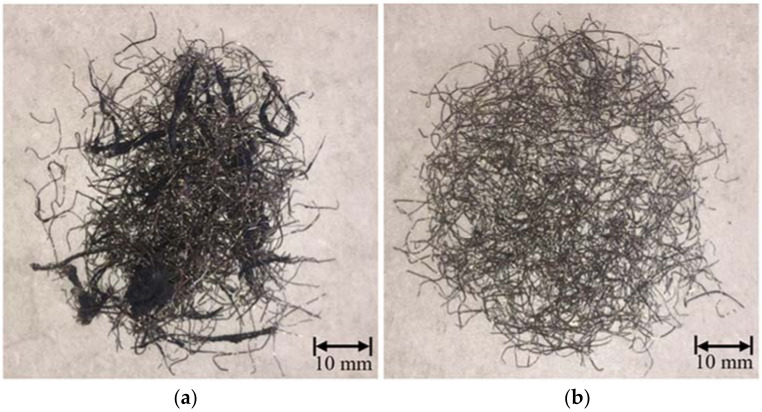
WTSF: (**a**) unclassified and (**b**) classified.

A local tire recycling company provided the residual steel fibers for the study. The tire recycling process includes grinding using pneumatic equipment with rotating shafts; this procedure scraps the tires into smaller particles. Subsequently, the residual steel fibers are removed using magnets. Shulman [47] describes the recycling process to obtain WTSF and rubber in detail. Since WTSF were mixed with residual rubber, a previous fibers material selection was made, as shown in Figure 3. The current study employed only the selected fiber for manufacturing soil–cement blocks (Figure 3b).

The steel fibers used had 23.22 ± 5.12 mm in length; Afroughsabet et al. [48] categorized this material as macro fibers based on its dimensions. Moreover, the diameter of the collected fibers was 0.23 ± 0.05 mm, such as those used by Pilakoutas et al. [9] and Hu et al. [49].

**Figure 4:**
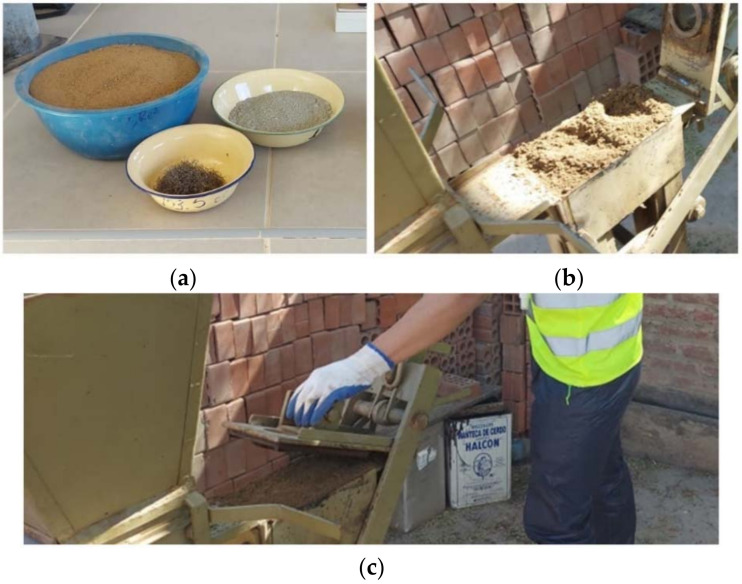
Preparation of soil–cement blocks: (**a**) preparation of materials, (**b**) placement of mixture, and (**c**) compaction.

**Figure 5:**
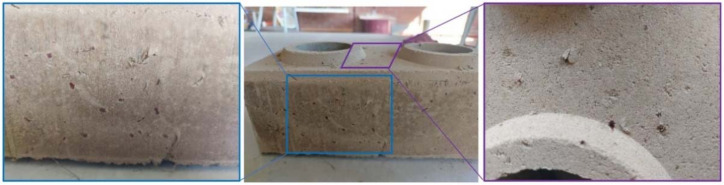
WTSF soil–cement block samples.

Soil–cement block samples were elaborated with a mechanical mixer and a manual Lego-type die press. Figure 4 shows the procedure followed to elaborate the soil–cement blocks. After the manufacturing process, the blocks were kept in the laboratory facilities for curing and drying for the next seven days. During this time, the block samples were isolated with plastic in their base to avoid rapid water loss, as recommended by Gatani [50]. Figure 5 depicts the soil–cement blocks incorporated with WTSF prior to the compression testing.

The blocks’ compressive strength was determined for 7, 14, and 28 days, utilizing four blocks per group age and residual steel fiber percentage.

The experimental methodology procedure followed the NBR 8492 standard [51] as the basis for conducting the test.

## 3. Results and Discussion

### 3.1. Soil Properties

**Figure 6:**
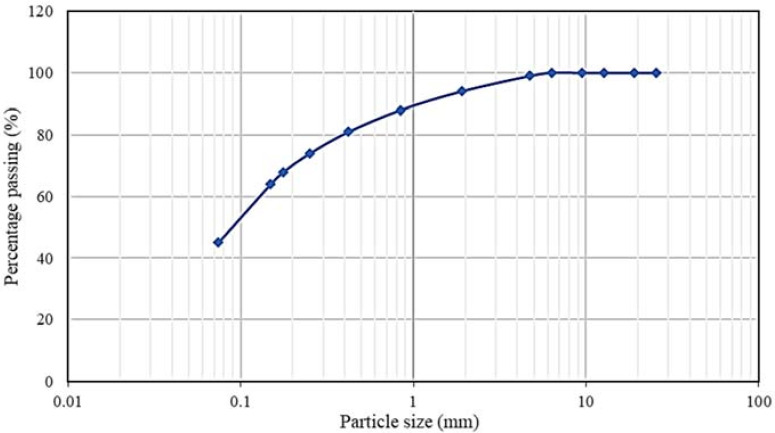
Soil’s granulometric classification curve.

Figure 6 depicts the results from the granulometric analysis of the soil. It was observed that the soil material was composed of sand (55%) and slime clay (45%).

Atterberg’s liquid limit (LL) and plasticity index (IP = LL − LP) obtained were 27 and 15, respectively. Therefore, the soil was classified as A-6 (i.e., clay soil) as per the AASTHO standard M145 [44] and clay sand (SC) according to the USCS [45] specification.

**Figure 7:**
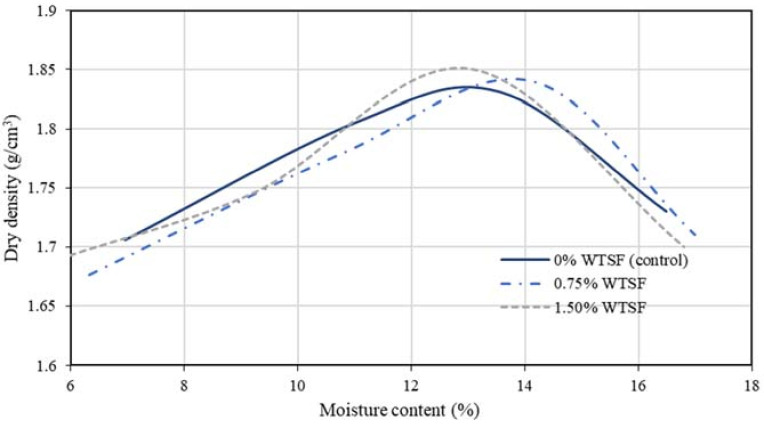
Proctor test’s compaction curves.

The Proctor test results are presented in Table 3 and Figure 7. Although the samples’ steel fibers’ addition showed independence from the optimum moisture content, the samples with steel had a higher dry density.

### 3.2. Compressive Strength

**Figure 8:**
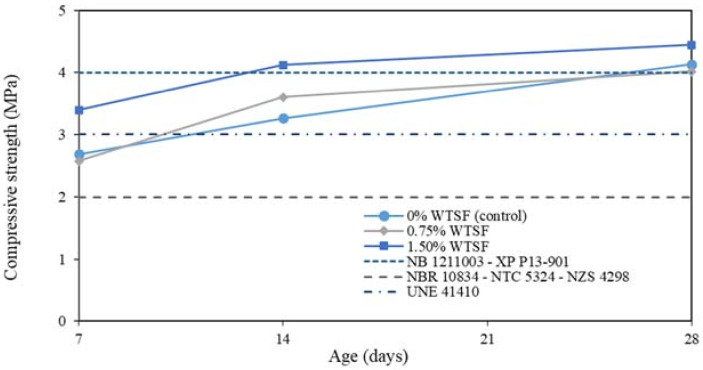
Block compression tests results.

Figure 8 depicts the compressive strength results of the tested blocks. Due to aging, an increase in compressive strength could be noticed, reaching a maximum value at 28 days. Moreover, the block samples with 1.50% WTSF presented the highest compressive strength values for all ages. However, WTSF’s effect at 0.75% showed a non-defined correlation with divergent values concerning the control sample (0% WTSF).

Soil–cement blocks’ compressive strengths shown in Figure 8 are satisfactory as per the Brazilian soil–cement standard [32]; they all laid above the minimum required of 2 MPa after seven days. Bolivia does not have a specific regulation for soil–cement blocks. However, the regulations of the Bolivian standard for ceramic bricks can be extended for this study [33]. Based on the latter, the specimens were typed as “Category C” with a minimum required compressive strength of 4 MPa for common materials required in building construction. Blocks with 1.50% WTSF showed good agreement with this category after 14 days of curing. The remaining blocks with 0% and 0.75% WTSF accomplished this requirement after 28 days of curing. Regarding the Colombian standard materials regulation [34], the soil–cement WTSF blocks met the minimum requirements of the “BSC-20” category (i.e., strength > 2 MPa). However, they could not reach the BSC-60 category with a minimum compressive strength required of 6 MPa.

Regarding the European regulations, the Spanish standard UNE 41410 [35] classifies compressed earth blocks according to their compressive strength: 1.3 MPa (BTC 1), 3 MPa (BTC 3), and 5 MPa (BTC 5). In this regulation, the usage of binders such as cement is optional. It was observed that all the soil–cement blocks with WTSF belonged to BTC 3 category after 14 days. Regarding the French standard XP P13-901 [36], the blocks reached the category BSC 40 (4 MPa) at 28 days. Finally, the New Zealand specification NZS 4298 [37] indicates a minimum compressive strength of 2 MPa, a value reached by all the tested samples after seven days.

These results are consistent with previous investigations on soil–cement blocks with steel fibers reinforcement that report improved mechanical properties [25,26]. Similarly, Nasir [52] and Ndyambaje [53] reported an enhancement in the compressive strength of cement-based materials reinforced with 1.5% WTSF and 1.2% WTSF, respectively; both authors employed recycled steel fibers of 40 mm in length. On the contrary, Mastali and Dalvand [54] demonstrated that the addition of lower percentages of WTSF (up to 0.75%) could also lead to positive outcomes regarding the compressive strength of cement-based materials.

Percentages used for WTSF in this study are similar to those reported in the literature [52,53,54]. However, these studies only consider concrete and mortar. The present study shows that these exact percentages of WTSF can also be extended for soil–cement blocks as a novel application of this tire residue. In this case, the soil–cement blocks with 1.5% WTSF have the most relevant results. Similarly, Eko et al. [25] found that 2% WSTF significantly improves the mechanical properties of unfired earth blocks. However, Eko et al. [25] used a lateritic soil and different block dimensions, which differs from the current study.

**Figure 9:**
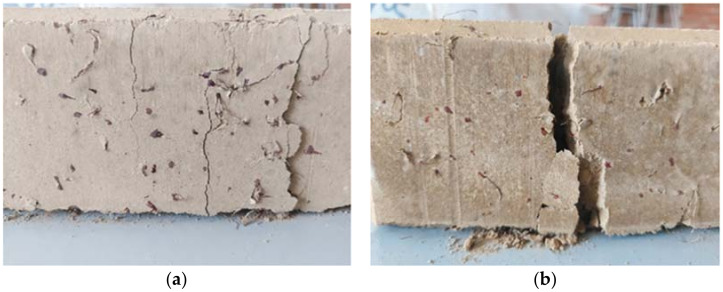
Tested soil–cement WTSF blocks: (**a**) 0.75% WTSF and (**b**) 1.50% WTSF.

Figure 9 below shows the soil–cement WTSF blocks after the compressive strength test.

In order to verify whether the compression strength average values by age are statistically significantly different, an analysis of variance (ANOVA) (Table 4) was performed, considering a significance value of 0.01.

To better understand Table 4, two hypotheses are proposed: null (H0), if F < F crit; there is not a significant difference between the means, and alternative (H1), if F > F crit, there is a significant difference between the means. As detailed in Table 4, F > F crit in all cases; therefore, there were statistically significant differences between the means, and all *p*-values were less than 0.01. Hence, it can be stated that there is a statistically significant difference between the block’s compressive strength average values ranked by age. In order to verify the latter, a Tukey test was carried out with a similar significance value (0.01). Thus, it seemed that there were no statistically significant differences between the means of the blocks with 0% WTSF and 0.75% WTSF (Table 5). Nevertheless, there were differences between these two percentages regarding the 1.50% blocks of WTSF. Therefore, the latter indicated that percentages greater than 1.50% of WTSF addition positively impact the compressive strength.

**Figure 10:**
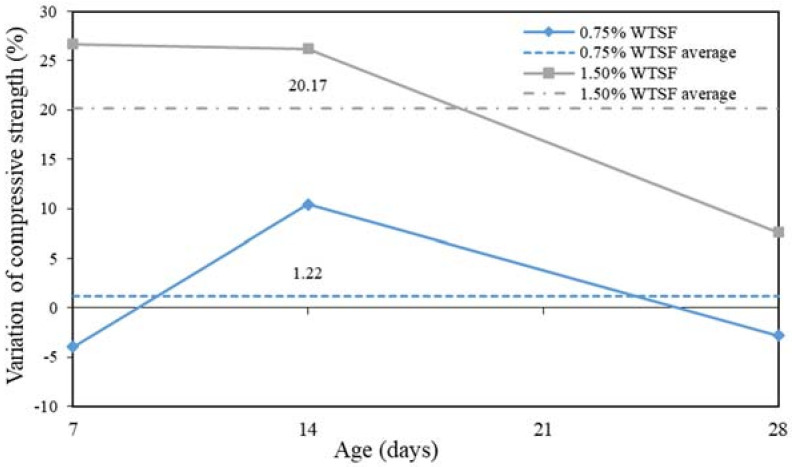
Percentage of variation in compressive strength.

Figure 10 shows the percentage of compressive strength variation compared to the control sample. As previously mentioned, the blocks with 0.75% of WTSF did not present statistically significant differences contrasted with the control samples. For this reason, an average variation of 1.22% was observed exclusively. Regarding the 1.50% blocks of WTSF, a positive average variation of 20.17% was detailed. However, the most significant growth in compressive strength occurred between 7 and 14 days of curing.

## 4. Conclusions

Based on the experimental test carried out in this study, the following conclusions can be drawn:

- The Proctor test on the samples revealed that the amount of WTSF does not influence optimal humidity content in the specimens. However, block samples with WTSF showed higher dry density.
- The highest compressive strength was found for the 1.50% WTSF blocks. This latter can be extended to all the samples regarding the content of WTSF in their matrices.
- After seven days of curing, all block samples topped the minimum compressive strength requirement established in the ABNT standard [32]. Regarding the Bolivian specification for building materials [33], the blocks were classified as Category C, accomplishing the minimum strength of 4 MPa after 14 days of curing for 1.50% WTSF blocks and after 28 days for 0.75% WTSF samples. At the age of 28 days, all the soil–cement blocks beat the minimum compressive strength requirements, considering the standards of Colombia [34], Spain [35], France [36], and New Zealand [37].
- An ANOVA analysis supported by the Tukey’s test on the compressive resistance’s results revealed that the growth in strength for soil–cement blocks tested occurred after seven days of curing and for 1.50 % WTSF blocks only. Thus, adding higher percentages of recycled steel fibers to the blocks may positively impact their compressive strength.
- The current study was exploratory, only considering the compressive strength assessment; the main mechanical property required by the regulations for its use in construction. Therefore, subsequent research is needed to consider other mechanical properties, such as tensile strength, flexural strength, durability, plastic energy absorption, large deformation resist, and others to consolidate the soil–cement WTSF blocks usage in building construction.
- The compressive strength results are favorable for 1.5% of WTSF, reaching the minimum category for construction with ease, especially for walls with nonstructural function. Thus, soil–cement blocks with WTSF are presented as a sustainable option in developing countries due to the reuse of waste and to help reduce the housing deficit.
- The current study was limited to three mixing groups, which does not provide a deep understanding of the influence of WTSF on the compressive strength of soil–cement blocks. Future lines of research may consider higher amounts of WTSF to find an optimal percentage addition to obtain advantageous physical and mechanical properties. Additionally, WTSF with rubber can be considered for further studies since this waste is obtained directly from the recycling companies. Hence, waste tire material selection could be avoided, saving time and providing greater practicality.
- Although this exploratory study accomplished the regulations regarding the minimum number of block samples to be tested by age, it is essential to explore larger samples to have more satisfactory results and have the possibility of a broader statistical analysis.

## Figures and Tables

**Table 1:** Chemical analysis for IP-30 cement.

Parameter	Unit	IP-30 Cement
Loss on ignition	%	2.33
SiO_2_	%	32.83
Al_2_O_3_	%	4.53
Fe_2_O_3_	%	2.32
CaO	%	50.77
MgO	%	4.55
SO_3_	%	2.10

Data provided by the manufacturer.

**Table 2:** Physical analysis for IP-30 cement.

Parameter	Unit	IP-30 Cement
Blaine	m^2^/kg	448
Residue T325	%	5.34
True Density	g/cm^3^	2.98
Bulk Density	g/cm^3^	1.05
Initial Setting	h	2.32
Final Setting	h	4.65
3-Day Strength	MPa	19.19
7-Day Strength	MPa	24.90
28-Day Strength	MPa	30.63

Data provided by the manufacturer.

**Table 3:** Proctor tests results.

Mixture	0% WTSF	0.75% WTSF	1.50% WTSF
Dry density (g/cm^3^)	1.83	1.84	1.85
Optimum moisture (%)	13.56	13.99	13.02

**Table 4:** ANOVA analysis for concrete strength delimited by aging.

Age	F	F Crit	*p* Value	Significance
7	53.94017	8.02152	9.75561E-06	Yes
14	39.49235	8.02152	3.50154E-05	Yes
28	16.04894	8.02152	0.00108	Yes

**Table 5:** Tukey’s test for concrete strength on an aging basis.

Group (WTSF)		*p* Value per Age (Days)		
		7	14	28
0%	0.75%	0.49364751	0.01659152	0.34869971
0%	1.50%	4.1885 × 10^−5^	2.6717 × 10^−5^	0.0078263
0.75%	1.50%	1.424 × 10^−5^	0.00124796	0.001025

## Data Availability

Not applicable.
